# Epidemiological and clinical characteristics of COVID-19 mortality: a retrospective study

**DOI:** 10.3389/fmed.2025.1464274

**Published:** 2025-03-10

**Authors:** Yaohua Hu, You Lu, Jiagui Dong, Delin Xia, Jin Li, Hong Wang, Min Rao, Chenxing Wang, Wanning Tong

**Affiliations:** ^1^Department of Respiratory and Critical Care Medicine, Naval Medical Center of People’s Liberation Army, Shanghai, China; ^2^Department of Respiratory Medicine, Shanghai Tenth People’s Hospital, Shanghai, China

**Keywords:** SARS-CoV-2, d-dimer, COVID-19, epidemiology, leukocyte

## Abstract

**Background:**

The global impact of SARS-CoV-2 and its associated coronavirus disease (COVID-19) has necessitated urgent characterization of prognostic biomarkers. This study aimed to delineate the epidemiological and clinical predictors of mortality among hospitalized COVID-19 patients.

**Methods:**

A retrospective cohort study was conducted on 123 patients with laboratory-confirmed COVID-19 admitted to Huoshenshan Hospital (Wuhan, China) from 1 February 2020 to 30 April 2020. Kaplan–Meier curve and multivariate Cox regression were used to assess the independent factors with survival time. Statistical significance was set at a *p*-value of <0.05.

**Results:**

The cohort exhibited a mortality rate of 49.6% (61/123), with the critical clinical type (HR = 7.970, *p* = 0.009), leukocytosis (HR = 3.408, *p* = 0.006), and lymphopenia (HR = 0.817, *p* = 0.038) emerging as independent predictors of reduced survival. Critical-type patients demonstrated significantly elevated inflammatory markers (neutrophils: 10.41 ± 6.23 × 10^9^/L; CRP: 104.47 ± 29.18 mg/L) and coagulopathy (D-dimer: 5.21 ± 2.34 μg/ml) compared to non-critical cases. Deceased patients exhibited pronounced metabolic derangements, including hyperglycemia (9.81 ± 2.07 mmol/L) and hepatic dysfunction (ALP: 174.03 ± 30.13 U/L).

**Conclusion:**

We revealed the epidemiological and clinical features of different clinical types of SARS-CoV-2 as summarized in this paper. We found that critical type, leukocyte, and lymphocyte are risk factors that affect survival time, which could be an early and helpful marker to improve management of COVID-19 patients.

## Introduction

The emergence of severe acute respiratory syndrome coronavirus 2 (SARS-CoV-2) in Wuhan, China in late 2019 marked the onset of a global pandemic, with its associated disease (COVID-19) triggering unprecedented public health challenges (1). Within weeks of the initial cluster of pneumonia cases (2), the World Health Organization (WHO) formally designated the outbreak of COVID-19 in February 2020 (3). Despite the Chinese government’s response rapidly to the epidemic and efforts to cut SARS-CoV-2 dissemination, including quarantining Wuhan city on January 23, since then, COVID-19 has swept and influenced the whole world. As of 15 November 2021, an estimated 254 million cases and 5.1 million deaths have been ascribed to COVID-19 (4). COVID-19 has changed the lives of human beings and severely affected all aspects of human life including health, economy, and culture.

Huang et al. (5) first reported clinical characteristics of 41 patients confirmed to be infected with COVID-19 on 2 January 2020, including 13 ICU patients and 28 non-ICU patients. Subsequent meta-analyses corroborated this clinical profile while quantifying symptom prevalence: fever (88.5%), cough (68.6%), fatigue (35.8%), and dyspnea (21.9%) (6). Therapeutic strategies remain predominantly supportive, with oxygen supplementation and mechanical ventilation constituting mainstays for critical care. The role of immunomodulators—particularly corticosteroids—has evolved through evidence from randomized trials, demonstrating mortality reduction when administered to hypoxic patients (7). In a prospective meta-analysis of clinical trials of critically ill patients with COVID-19, administration of systemic corticosteroids was associated with lower 28-day all-cause mortality (8). Although COVID-19 infection has now become normal, the epidemic has left a profound impact on human history. Mankind has already confronted multiple outbreaks of coronavirus such as severe acute respiratory syndrome (SARS), and Middle Eastern Respiratory Syndrome (MERS). No one knows whether a similar outbreak would occur again. We should learn enough from this outbreak to deal with the challenges ahead. For this reason, the characteristics of the virus must be well known. Some biological indicators, such as white blood cells, D-dimer, and C-reactive protein increased correspondingly in other serious bacterial infections. We hypothesize that the indicators above earlier identify the severity of infection and predict the outcome and discovered some other indicators. Thus, the purpose of this article is to summarize the clinical features, symptoms, and complications of COVID-19 patients, especially dead ones in Huoshenshan Hospital, which was responsible for treating critical patients in Wuhan, from February to April in order to help provide clinical information in the management of this outbreak.

## Methods

### Data source

We performed a retrospective study focusing on the epidemiological and clinical characteristics of patients diagnosed with COVID-19 pneumonia from 1 February 2020 to 30 April 2020. All patients were assigned to Huoshenshan Hospital in Wuhan. The Huoshenshan Hospital took 10 days from design to completion. The Chinese People’s Liberation Army selected 1,400 medical personnel to undertake the medical treatment of critical patients diagnosed with pneumonia caused by COVID-19 in Wuhan from 3 February 2020. The data were uniformly collected by the Chinese People’s Liberation Army Naval Medical Center. This retrospective study was approved by the Chinese People’s Liberation Army Naval Medical Center (ID: 2024108) and performed following the Helsinki Declaration of 1964 (revised 2008). Informed consent was not required of the patients.

### Diagnosis

All the patients were diagnosed with COVID-19 based on the WHO interim guidance in this study. The subtype of COVID-19 patients was defined according to the Chinese diagnosis and treatment scheme for SARS-CoV-2 (seventh edition) with minor modifications based on the WHO standards. Disease severity was classified per Chinese Clinical Guidelines (seventh edition): Common: Radiographically confirmed pneumonia without hypoxia; severe: respiratory rate ≥ 30/min, SpO₂ ≤93%, or PaO₂/FiO₂ <300 mmHg; Critical: Mechanical ventilation requirement, shock, or multiorgan failure.

### Procedures

We obtained epidemiological, clinical, laboratory, treatment, and outcome data from the medical records. Clinical outcomes were followed up to 30 April 2020. If data were missing, we obtained data from the attending doctors. All data were checked by at least two doctors. SARS-CoV-2 infection was confirmed by real-time reverse transcription-polymerase chain reaction (RT-PCR) using the protocol described previously. Other laboratory examinations were conducted in the Huoshenshan Hospital. All patients underwent chest X-rays or chest computed tomography (CT) during the hospitalization.

### Outcomes

All the patients were either discharged or died after the treatment. We described and summarized the clinical data including epidemiological anthropometrics, symptoms and signs on admission, laboratory examination, comorbidity, treatment (including drugs, intensive care, and mechanical ventilation) and clinical outcomes.

### Statistical analysis

Continuous variables were expressed as mean ± SD or median (IQR). Intergroup comparisons utilized ANOVA with Tukey’s post-hoc test. Survival analysis used Kaplan–Meier curves with log-rank testing. Multivariate Cox proportional hazards regression incorporated variables with a *p*-value of <0.1 in univariate analysis. Statistical significance was defined as a two-tailed *p*-value of <0.05. SPSS (version 24.0) was used for all analyses.

## Result

### Demographic characteristics

This study analyzed 123 patients diagnosed with COVID-19 pneumonia from 1 February 2020 to 30 April 2020 in the Huoshenshan Hospital in Wuhan. The cohort (mean age 66.2 ± 12.4 years; 57.7% male) demonstrated high comorbidity prevalence (49.6%). Only one patient reported direct exposure to the presumed zoonotic origin (South China Seafood Market), while 33 patients (26.8%) had confirmed contact with COVID-19 cases (Table 1). A total of 65% of patients had at least one coexisting medical history. The top three of these medical histories were hypertension, diabetes, and chronic liver disease.

**Table 1:** Demographic characteristics of 123 patients with SARS-CoV-2 infection in the Huoshenshan Hospital.

	*N* (%)
Gender	
Male	71 (57.7)
Female	52 (42.3)
Age (average = 66.18)	
<60	40 (32.5)
60 ~ 80	65 (52.8)
>80	18 (14.6)
Exposure History	
South China Seafood Market	1 (0.8)
Contact with patients	33 (26.8)
History	
Any disease	80 (65.0)
Hypertension	49 (39.2)
Diabetes	31 (24.8)
Coronary heart disease	16 (12.8)
Cerebrovascular disease	15 (12.0)
Nephritis	15 (12.0)
Blood disease	11 (8.8)
Tumor	9 (7.2)
Liver disease	3 (2.4)

### Clinical and laboratory characteristics

The clinical characteristics of the patients are shown in Table 2. Fever (73.2%), fatigue (52.0%), and cough (38.2%) represented the predominant presenting symptoms, with rare neurological manifestations (dizziness/vomiting <5%). The radiographic assessment revealed ground-glass opacities in 82.1% of cases, predominantly involving ≤2 pulmonary lobes (74.8%). The rest patients had multilobe involvement, which may mean a more serious illness. As to their outcome, 61 patients died during the hospitalization.

**Table 2:** Clinical characteristics of 123 patients with SARS-CoV-2 infection in the Huoshenshan Hospital.

	*N* (%)
Clinical type	
Common type	64 (52.0)
Severe type	24 (19.5)
Critical type	35 (29.5)
Symptoms	
Fever	90 (73.2)
Fatigue	66 (52.0)
Cough	47 (38.2)
Muscle ache	40 (32.5)
Difficulty breathing	30 (24.4)
Respite	24 (18.9)
Decreased appetite	21 (16.5)
Diarrhea	10 (7.9)
Chills	5 (3.9)
Dizziness	2 (1.6)
Vomiting	2 (1.6)
CT manifestations	
ground glass	101 (82.1)
Cord shadow	17 (13.8)
Consolidation	12 (9.8)
Pleural effusion	5 (4.1)
Hollow	1 (0.8)
Number of diseased lung lobes	
1–2	92 (74.8)
3–5	31 (25.2)
Outcomes	
Discharge	62 (50.4)
Died	61 (49.6)

By definition of clinical type, 64 patients (52.0%)were diagnosed as common type. Twenty-four patients (19.5) were diagnosed as severe type, as shown in Table 3. The rest was diagnosed as critical type. The average age in critical type was 72.92 years, older than the other groups. However, they had the shortest hospital stay. The time it takes for the nucleic acid to turn negative for the first time was 13.71, 17.00, and 23.29 in the three-type group. In the routine blood test, patients of critical type had higher white blood cell counts, including highest neutrophils, lowest lymphocytes, lowest proportion of monocytes, and highest eosinophils but lowest proportion of eosinophils. Patients of critical type had lower platelets and higher c-reactive protein. In terms of liver function indicators, patients of critical type have lower albumin, higher fasting glucose, and high alkaline phosphatase.

**Table 3:** Clinical and laboratory characteristics among the three clinical types.

	Common type (*n* = 64)	Severe type (*n* = 24)	Critical type (*n* = 35)	*p*-value
Age	61.22	69.29	72.94	0.001*
Temperature	36.62	36.92	36.97	0.24
HR	82.91	92.62	96.20	0.001*
R (without oxygen)	20.34	21.29	26.29	0.001*
SBP	129.97	132.50	133.31	0.657
DBP	79.00	76.50	77.60	0.660
SPO_2_ (oxygen inhalation)	88.33	92.20	92.89	0.466
Median survival time	23	21	9	0.001*
Median of hospital stay	15	16	9	0.516
Median time from onset to death	21	37	27	0.608
COVID-19 negative for the first time	13,71	17.00	23.29	0.012*
Hb	124.21	121.33	122.75	0.910
RBC	4.03	4.23	3.94	0.613
WBC	5.90	8.60	11.47	0.000*
N	4.61	7.40	10.41	0.000*
N%	48.78	23.32	50.55	0.33
L	1.54	0.94	0.78	0.014*
L%	24.19	2.22	15.39	0.000*
M	0.51	11.13	6.95	0.000*
M%	7.48	1.95	0.48	0.000*
E	0.14	2.38	4.78	0.000*
E%	1.97	0.15	0.58	0.000*
PLT	254.10	275.33	166.69	0.003*
CRP	18.74	91.22	104.47	0.000*
PCT	0.75	0.84	1.84	0.582
FBG	5.42	9.88	9.14	0.000*
ALT	40.31	29.46	42.75	0.507
AST	29.79	23.88	46.91	0.014
ALP	72.77	78.37	110.59	0.000*
GGT	43,46	55.26	188.76	0.072
TB	8.02	21.00	16.00	0.001*
DB	2.88	9.97	8.11	0.000*
IB	4.80	14.48	8.71	0.037*
TBA	7.56	6.23	4.46	0.788
ALB	36.07	32.96	31.43	0.001*
GLB	28.18	29.01	28.07	0.901
A/G	1.31	1.16	1.15	0.014*
LDH	218.79	403.18	511.15	0.0001*
CK	60.80	175.98	139.04	0.018*
CK-MB	13.47	20.68	21.21	0.147
BNP	89.43	248.75	486.98	0.000*
Mb	36.54	63.24	112.30	0.357
PT	14.42	14.65	16.46	0.377
APTT	28.27	29.89	30.83	0.204
INR	1.21	1,22	1,37	0.424
FD	3.06	3.70	2.64	0.02*
DD	2.36	7.26	4.84	0.02*
Cr	76.80	73.89	152.87	0.086
BUN	5.25	8.08	12,25	0.001
UA	259.71	229.20	317.07	0.195
K^+^	4.56	4.40	4.64	0.782
Na^+^	141.45	152.15	141.55	0.007*
Cl^−^	105.86	117.75	105.65	0.004*
Ca^2+^	2.11	2.05	1.92	0.000*

HR, heart rate; R, respiratory rate; SBP, systolic blood pressure; DBP, diastolic blood pressure; SPO_2_, pulse oxygen saturation; COVID-19 negative for the first time, the time for the nucleic acid to turn negative for the first time; Hb, hemoglobin; RBC, red blood cell; WBC, white blood cell; N, neutrophil; N%, neutrophil percentage; L, lymphocyte; L%, lymphocyte percentage; M, monocyte; M%, monocyte percentage; E, eosinophils; E%, eosinophils percentage; PLT, platelets; CRP, c-reactive protein; PCT, procalcitonin; FBG, fasting blood glucose; ALT, alanine aminotransferase; AST, aspartate aminotransferase; TB, total bilirubin; DB, direct bilirubin; IB, indirect bilirubin; TBA, total bile acid; ALP, alkaline phosphatase; ALB, albumin; GLB, globulin; GGT, γ-glutamyl transpeptidase; LDH, lactate dehydrogenase; CK, creatine kinase; CK-MB, creatine kinase-MB; BNP, brain natriuretic peptide; Mb, myoglobin; PT, prothrombin time; APTT, activated partial thromboplastin time; INR: international normalized ratio; DD, d-d dimer; Cr, creatinine; BUN, blood urea nitrogen; UA, uric acid; K^+^, potassium ion; Na^+^, sodium ion; Cl^−^, chloride; Ca^++^, calcium ion. *p < 0.05.

As shown in Table 4, we divide into two groups based on prognosis. A total of 61 patients died during the hospitalization, while 62 patients were discharged eventually. The average age of dead patients was 71.62 years, older than the other group. The length of stay in the two groups was almost similar. In the routine blood test, dead patients had higher white blood cell counts, including higher neutrophils and lower lymphocytes. Patients in the dead group had higher c-reactive protein and higher procalcitonin. In terms of liver function indicators, the dead patients had higher values in fasting glucose, alkaline phosphatase, and γ-glutamyl transpeptidase. Patients in the dead group had more value of d-dimer. Creatine kinase, lactate dehydrogenase, and α-hydroxybutyrate dehydrogenase were much higher in dead groups. The brain natriuretic peptide index in the dead group was higher. There is no significant difference in the renal function indexes of the patients.

**Table 4:** Clinical and laboratory characteristics between the two outcome groups.

	Discharge (*n* = 62)	Died (*n* = 61)	*p*-value
Age	60.74	71.61	0.128
Length of hospital stay	14.38	14.74	0.000*
COVID-19 negative for the first time	14.07	20.36	0.014*
Hb	124.42	121.95	0.135
RBC	4.02	4.01	0.343
WBC	5.81	10.46	0.000*
N	4.46	9.39	0.005*
N%	51.75	37.90	0.000*
L	1.63	0.75	0.012*
L%	25.26	10.06	0.169
M	0.51	7.41	0.000*
M%	7.41	0.89	0.014
E	0.21	4.46	0.000*
E%	1.97	0.79	0.001*
PLT	259.08	170.47	0.627
CRP	15.03	107.21	0.0001*
PCT	0.14	1.72	0.021*
FBG	5.49	9.807	0.000*
ALT	39.31	39.67	0.714
AST	28.00	41.71	0.096
ALP	73.21	174.03	0.007*
GGT	42.06	160.11	0.012*
TB	7.91	4.03	0.001*
DB	2.76	8.76	0.000*
IB	5.09	9.37	0.042*
TBA	7.67	4.54	0.313
ALB	37.01	30.94	0.902
GLB	27.98	28.53	0.378
A/G	1.37	1.10	0.775
LDH	207.88	513.82	0.000*
α-HBDH	169.58	402.18	0.000*
CK	67.66	143.20	0.004*
CK-MB	12.17	21.59	0.16
BNP	63.06	426.07	0.000*
PT	13.80	16.32	0.854
APTT	28.92	29.92	0.522
INR	1.16	1,36	0.803
DD	1.62	6.19	0.005*
Cr	99.59	94.47	0.417
BUN	4.88	10.79	0.000*
UA	272.71	263.25	0.455
K^+^	4.57	4.58	0.059
Na^+^	141.54	142.46	0.000*
C^−^	105.68	107.34	0.000*
Ca^2+^	2.12	1.91	0.176
Mg^2+^	0.99	0.99	0.19

COVID-19 negative for the first time, the time for the nucleic acid to turn negative for the first time; Hb, hemoglobin; RBC, red blood cell; WBC, white blood cell; N, neutrophil; N%, neutrophil percentage; L, lymphocyte; L%, lymphocyte percentage; M, monocyte; M%, monocyte percentage; E, eosinophils; E%, eosinophils percentage; PLT, platelets; CRP, c-reactive protein; PCT, procalcitonin; FBG, fasting blood glucose; ALT, alanine aminotransferase; AST, aspartate aminotransferase; TB, total bilirubin; DB, direct bilirubin; IB, indirect bilirubin; TBA, total bile acid; ALP, alkaline phosphatase; ALB, albumin; GLB, globulin; GGT, γ-glutamyl transpeptidase; LDH, lactate dehydrogenase; α-HBDH, α-hydroxybutyrate dehydrogenase; CK, creatine kinase; CK-MB, creatine kinase-MB; BNP, brain natriuretic peptide; Mb, myoglobin; PT, prothrombin time; APTT, activated partial thromboplastin time; INR, international normalized ratio; DD, d-d dimer; Cr, creatinine; BUN, blood urea nitrogen; UA, uric acid; K^+^, potassium ion; Na^+^, sodium ion; Cl^−^, chloride; Ca^++^, calcium ion. *p < 0.05.

### Outcomes

**Figure 1:**
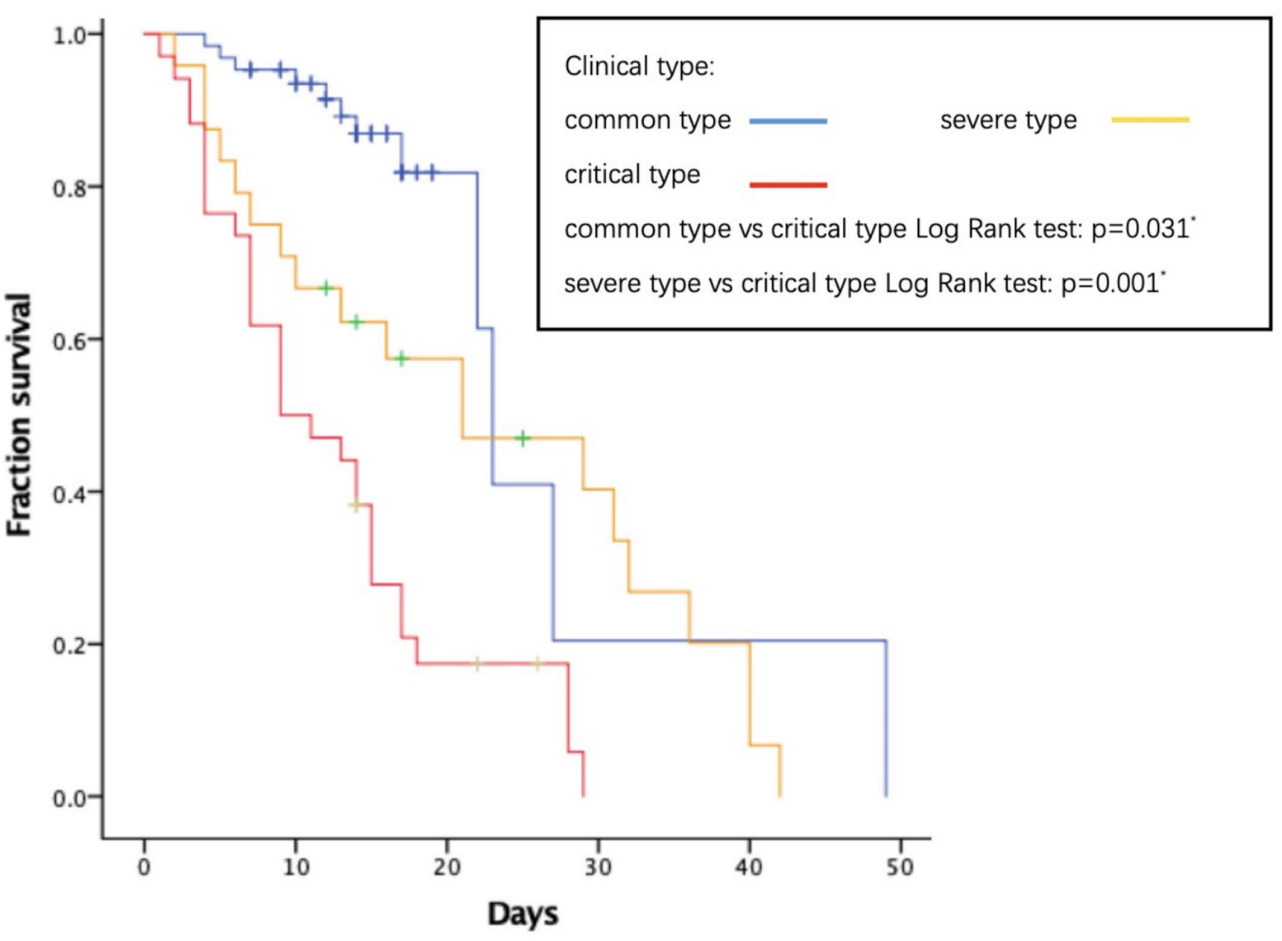
Relationship between clinical type and survival time.

**Figure 2:**
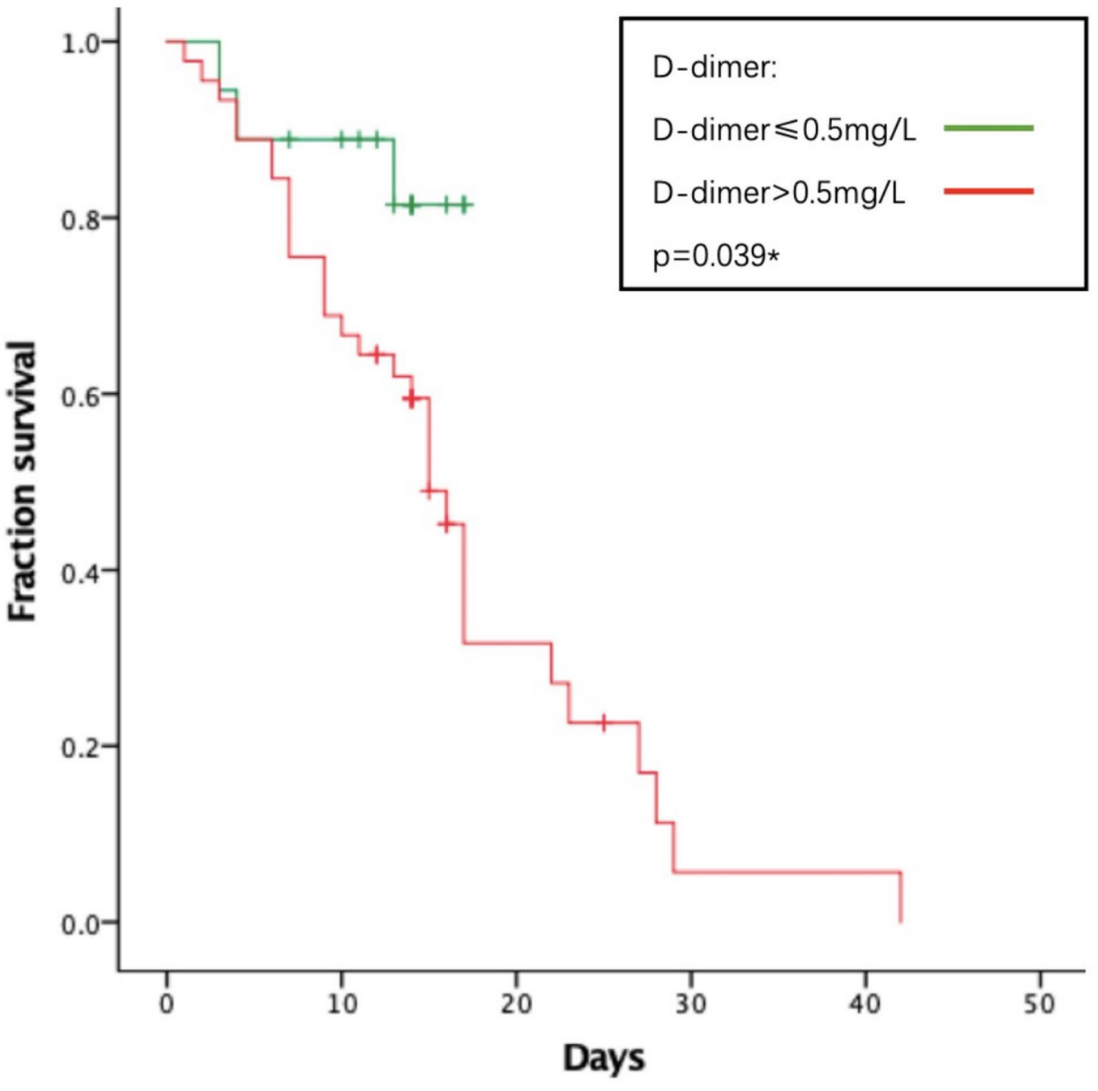
Relationship between d-d dimer and survival time.

**Figure 3:**
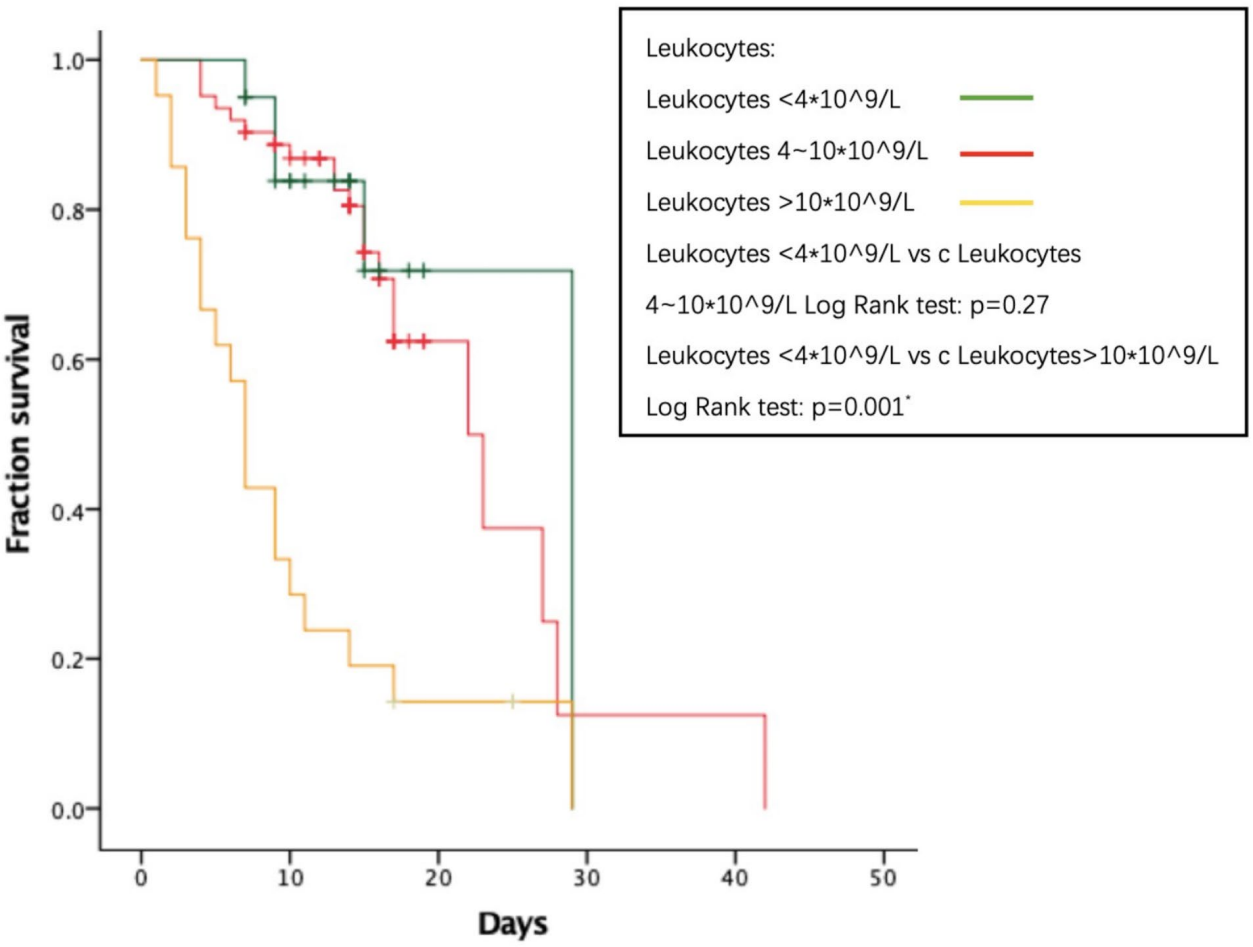
Relationship between leukocytes and survival time.

**Figure 4:**
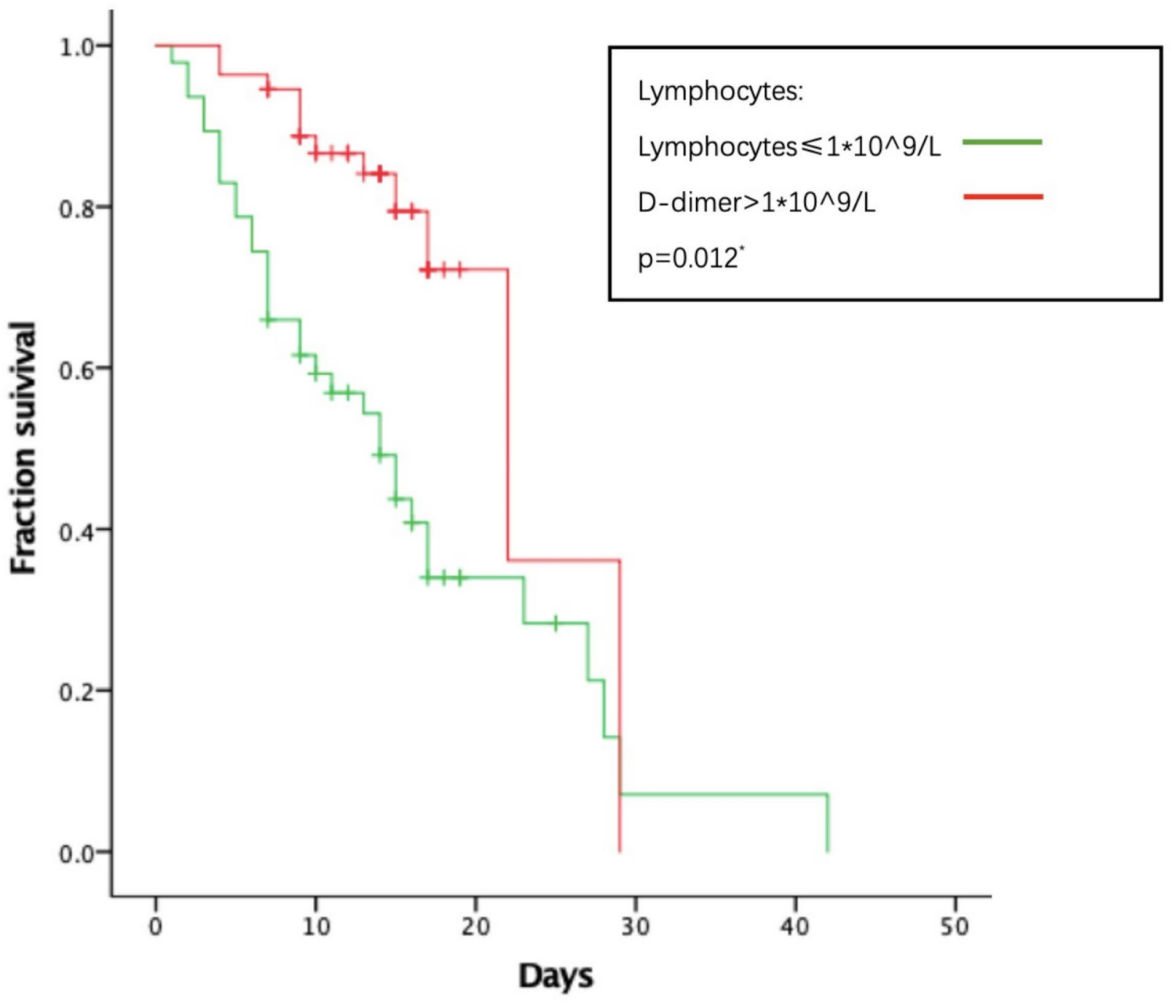
Relationship between lymphocytes and survival time.

**Figure 5:**
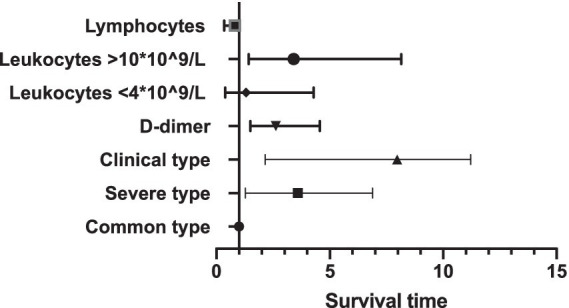
Associations of the actors with survival time.

As to their outcome, 61 patients died during the hospitalization. The average length of hospital stay was 14.74 days. The average time from onset to death is 31.78 days. We analyzed several factors related to patients’ survival time as shown in Figures 1–4. As shown in Figure 1, patients of critical type survived obviously shortly than those of the other clinical types (*p* = 0.015). The median survival time in the common group, severe group, and critical group was relatively 23, 21, and 9 days. Within 20 days after admission, the mortality rate of common patients was very low. However, 20 days after admission, the mortality rate of mild patients increased significantly than before. The survival time of the patients with increased d-dimer was 15 days, as shown in Figure 2 (*p* = 0.039). The counts of white blood cells and lymphocytes in blood routine indicators are also correlated with survival time. The survival time of patients with decreased leukocytes (median = 22) was shorter than the normal leukocytes (median = 29), while the survival time of increased leukocytes was shortest (median = 9), as shown in Figure 3 (*p* = 0.001). Figure 4 illustrates that the median survival time of patients with decreased lymphocytes (median = 14) was shorter than the normal lymphocytes (median = 22) (*p* = 0.012). We also analyzed the correlation of the factors above with the survival time. It turned out that the clinical type significantly increased leukocytes and decreased lymphocytes affected survival time, as illustrated in Table 5 and Figure 5.

**Table 5:** Associations of the actors with survival time.

	HR (95%CI) for PFS	*p*
Clinical group		
Common type	1.00 (ref.)	
Severe type	3.59 (1.280, 6.880)	0.029^*^
Critical type	7.970 (2.153, 11.216)	0.009^*^
D-dimer		
D-dimer≤0.5 mg/L	1.00 (ref.)	
D-dimer>0.5 mg/L	1.320 (0.150, 2.561)	0.50
Leukocytes		
Leukocytes 4 ~ 10*10^9/L	1.00 (ref.)	
Leukocytes <4*10^9/L	1.306 (0.398, 4.285)	0.660
Leukocytes >10*10^9/L	3.408 (1.424, 8.153)	0.006^*^
Lymphocytes		
Lymphocytes≤1*10^9/L	1.00 (ref.)	
Lymphocytes>1*10^9/L	0.817 (0.344, 0.938)	0.038^*^

Data were hazard ratio (95% confidence interval).

* Statistical significance at a *p*-value of < 0.05.

## Discussion

SARS-CoV-2 is a virion with approximately 200 nm diameter and a single positive-sense RNA genome (9). This retrospective cohort study delineates the epidemiological and pathophysiological determinants of mortality among 123 critically ill COVID-19 patients treated at Huoshenshan Hospital—a dedicated tertiary care center during Wuhan’s initial outbreak. We found that the patients in Huoshenshan Hospital were older and had more comorbidities and mortality than those reported by Zhou and Yu et al. (10) first in Wuhan. The advanced age and high mortality rate may be due to the serious condition of the patients admitted to the Huoshenshan Hospital. The findings in this series of patients suggest that SARS-CoV-2 can cause severe illnesses such as ARDS and shock (11). The most common signs of COVID-19 are fever, headache, cough, shortness of breath, and myalgia (12). Although the primary target for SARS-CoV-2 in the human body is the lungs, disease progression is associated with multiorgan injury by severely affecting the kidney and heart (13). Since 20–25% of patients with SARS-CoV-2 infection are known to have diarrhea (14), the change in digestive symptoms may be due to the virus mutation leading to an increased affinity for the gastrointestinal tract (15, 16).

The patients were divided into three clinical type groups according to the severity of COVID-19 was categorized as common, severe, or critical. The critical patients were older and had worse vital signs on admission. The positive nucleic acid meant that the virus was still active, which is closely related to the prognosis (17). Critical patients usually had other organ damage, mainly manifested in liver, heart, and blood coagulation functions. Cardiac complications, including heart failure, arrhythmia, or myocardial infarction are common in patients with pneumonia (10). The presence of abnormal liver tests and liver injury were associated with the progression to severe pneumonia (18). The detrimental effects on liver injury were related to certain medications, such as lopinavir/ritonavir, used during hospitalization (19). Coagulopathy is common in critically ill patients with COVID-19. Studies have reported an increase in D-dimer and fibrinogen concentrations in the early stages of COVID-19 disease (20).

Our study highlights the critical role of clinical types, leukocyte dynamics, lymphopenia, and elevated d-dimer in predicting mortality among COVID-19 patients. These factors are not merely prognostic markers but are deeply intertwined with the pathophysiological mechanisms driving severe outcomes. Severe and critical cases were characterized by hyperinflammation and multiorgan dysfunction, consistent with prior reports (14). The transition from mild to critical disease is often mediated by a cytokine storm, marked by excessive production of IL-6, TNF-α, and IL-1β, which amplify endothelial injury and promote ARDS (21). Furthermore, severe cases exhibit higher viral persistence in the lower respiratory tract, leading to prolonged immune activation and tissue damage (10). These mechanisms collectively explain why patients with critical clinical types face a significantly higher risk of death. Elevated leukocyte counts, particularly neutrophilia, were strongly associated with mortality in our cohort. Neutrophils in severe COVID-19 contribute to lung injury through two key mechanisms: Reactive oxygen species (ROS) and protease release and neutrophil extracellular traps (NETs). Activated neutrophils release ROS and proteases (e.g., elastase), directly damaging alveolar epithelial cells and exacerbating pulmonary edema (22). NETs promote immunothrombosis by trapping platelets and red blood cells, occluding microvasculature in the lungs and kidneys (23). This neutrophilic dominance reflects a maladaptive immune response, where hyperinflammation outweighs viral clearance, driving organ failure. Lymphopenia emerged as a hallmark of fatal outcomes, aligning with global studies (24). The depletion of CD4+ and CD8+ T cells compromises antiviral immunity, allowing unchecked viral replication (25). As to the higher index d-dimer, the dead patients had severe dysfunction in blood coagulation function. Viral invasion of endothelial cells triggers tissue factor expression, activating the extrinsic coagulation pathway (26). Furthermore, fibrin deposition in pulmonary and renal vasculature leads to ischemic organ damage, while macrothrombotic events (e.g., pulmonary embolism) further worsen hypoxia (27). Systemic microvascular thrombosis may occur in most deaths (28, 29). In summary, clinical type, leukocyte, lymphocyte, and d-d dimer are risk factors that affect survival time, which could be an early and helpful marker to improve the management of COVID-19 patients. Not only pathophysiological effects of SARS-CoV-2 but also pre-existing comorbidities and therapeutic interventions during hospitalization significantly influence survival outcomes in COVID-19 patients. Hypertension, diabetes, and cardiovascular diseases emerged as critical determinants of mortality. A variety of treatments significantly modulated survival outcomes, including antivirus therapy and respiratory support. These factors that affect prognosis require further research in the future.

There are several limitations of this study that need to be acknowledged. First, the study was retrospective, which may decrease its credibility and future prospective cohort studies. Second, the sample size was small, which may lead to a lack of universality. Finally, there were other factors that affect prognosis such as treatment and pre-existing comorbidities which had been reported in a previous report. Therefore, it would require further research.

## Conclusion

In summary, we revealed the epidemiological and clinical features of different clinical types of SARS-CoV-2 as summarized in this paper. We also found that leukocyte, lymphocyte, and clinical type are risk factors that affect survival time, which could be an early and helpful marker to improve the management of COVID-19 patients. Further in-depth analysis of the therapeutic interventions is under way.

## Data Availability

The original contributions presented in the study are included in the article, further inquiries can be directed to the corresponding author.
